# Supplementary material for: Psychological distress and burden of care among family caregivers of patients with mental illness in a neuropsychiatric outpatient clinic in Nigeria
Source: PLoS One. 2021 May 6;16(5):e0250309. doi: 10.1371/journal.pone.0250309 (PMC8101711; doi:10.1371/journal.pone.0250309)
Supplement: Attachment — Submitted filename: Response to reviewer.docx [file pone.0250309.s001.docx]

**REBUTAL LETTER**

Dear Editor,

Following the review of our manuscript by PLOS and the comments made from the reviewers, the authors express their appreciation to the reviewers for their well-articulated views and criticism of our manuscript. The comments made by the reviewers have helped to further improve the manuscript.

Comments were made by two reviewers. However, only comments from Reviewer #2 raised issues with the manuscript, and we have attempted to address all the issues raised by the reviewer. The authors hereby give their point-by-point response to the queries by the reviewer in the following section. Author’s responses are marked in blue text.

Warm regards

Ekerettte Udoh

……………………………………………………………………………………………………………………………………….

**Reviewer #2**: The manuscript tackles one very relevant point, that is the psychological distress and burden of care of relatives of people suffering from mental illness, in a low/middle-income country where the rates of psychiatric patients accessing specialistic cares are disproportionately low compared to the needs of these individuals.
Despite this extremely valuable starting point, unfortunately the study design has some limitations and weakness that constraints me, although reluctantly, to propend for the rejection of the manuscript. Nonetheless, given the high social merits of the research, I'd rather indicate major revision, also to integrate my comments with those of the other reviewers and to allow Authors to respond my criticisms and, if they would be willing, to try to fix some issues I will raise.

I see three major limitations/weakness in the study.
1. The inclusion of heterogeneous psychiatric and even neurological (epilepsy) conditions. This fact biases the rates of psychological distress and burden of care among participants. Authors should have better done to focus only on one or a restricted number of conditions (possibly severe ones) or compare, if they had some rationale for doing it, distress/burden in one condition compared to another (for instance: one severe psychiatric diagnosis compared to another one or to a severe medical disorder)

***Author Response****: The reviewer’s observation on the design of the study employing a heterogenous psychiatric condition is very valid. The authors have agreed with the reviewer’s suggestion to include it as a limitation of the study. Also, the points raised that study would have better focused on a restricted number of conditions or to compare distress and burden in different conditions, or another medical disorder has been given as recommendations for further study in the manuscript.*

2.50.4% of participants had a higher education degree. Is this rate consistent with education level in the general population? It will not in many western countries. I also noticed that more than 60% participants were in the higher monthly earning category. Why the rate of higher education participants was so high? Was this related to the kind of hospital where recruitment took place? Or to the geographical localization of the site (in a richest region of Nigeria, for instance)? Should this represent a challenge for generalization of findings to the Nigerian general population?

***Author Response****: The rates of higher educational qualification found in our study is very plausible, given that the study was carried out in an urban area in Nigeria, and the facility is a tertiary hospital. We found other studies with similar high educational qualification. In a tertiary hospital-based study in an urban setting in Lagos up to 48% participants reported higher educational qualification^[1]^.*

*Regarding ‘monthly earnings’, in our study we adopted the highest monthly earning category as the earnings above the national minimum income, which is N30,000 (which is about $65). Therefore, the high proportion (60%) in the highest monthly earning is plausible as many people earn more than the minimum wage of N30,000. We also see that a large proportion also earn less than the minimum wage, which is typical of the population.*

*Notwithstanding, the authors have emphasized caution on the generalization of the findings to the general Nigeria population.*

3. Only caregivers of psychiatric patients were included. While this allowed to capture the level of psychological distress and burden of care in this group of people, it adds nothing to the comprehension of how severe are distress and burden relatively to other kinds of medical disorders.

***Author Response****: The observation by the reviewer is very valid. We have cited a literature presenting rate of caregiver’s burden and psychological distress in a non-psychiatric medical condition in Nigeria for reference and comparison for their rates with findings of our study.*

Also, no comparison is given with other regions of the country, with other African or less-developed regions, or with well-developed countries. Therefore, the study only gives a photography of what happens in a limited geographical region, but it does not allow to locate the findings in a broader and more meaningful context, if not with indirect comparisons.

***Author Response****: In the discussion section of our manuscript some evidence from studies in other developed and developing regions as reference for comparison with our findings have been provided.*

Additional comments.
1. Authors should indicate additional inclusion/exclusion criteria (if any) beyond the 18-65 age range. For instance, were relatives with mental disorders included? All type of relatives were included or only those who spent considerable daily or weekly time with the patient (just to figure out a possibility: once the patient was assisted at the appointment by a brother/sister not living with him/her, was the relative recruited?).

***Author Response****: Two additional exclusion criteria that we employed have been included in the manuscript.*

The 18-65 age range may be too stringent. A 70-year old father of 40-50-year old patient would have been excluded, but his psychological distress and burden of care may be expected to be extremely severe.

***Author Response****: This is a valid observation. We are, however, not able to make any revision in this regard.*

2. Authors should specify the dates when the recruitment was conducted

***Author Response****: The dates of data collection have been added in the method section.*

3. In the section "Background characteristics of respondents" what "status of number of children" is?

***Author Response****: The variable that shows the ‘status of number of children’ has been added in the descriptive and bivariate association results. In our initial analysis this variable was dropped, because we rather considered use of the variable ‘’Having children.’’*

4. "When the factor loadings for the components were greater than 0.3" I believe Authors would intend lower than 0.3 in this point.

***Author Response****: This has been corrected to now read as ‘’lower than 0.3.’’*

5. There are some grammar imprecisions, such as "Any value greater than 0.30 are". Authors should carefully revise English language

***Author Response****: The article has been carefully revised and some imprecise grammars have been corrected.*

indicated
6. Factor analysis adds very few to the findings.

***Author Response****: The components from the factor analysis were numerous, thus, the authors limited their discussion to keep the article less lengthy.*

7. Discussion is too long. The Limitations paragraph accounts only for some potential biases and does not discuss the relevant limitations I have above

***Author Response****: Some less relevant points in the discussion section have been removed. We have also accepted all suggestions to include the study limitations raised by the reviewer.*

1. A. J. Ogunmodede, O. Abiodun, A. B. Makanjuola, J. K. Olarinoye, J. A. Ogunmodede, and O. I. Buhari, “Burden of Care and Psychological Distress in Primary Caregivers of Patients with Type -2 Diabetes Mellitus in A Tertiary Hospital in Nigeria,” *Ethiop. J. Health Sci.*, vol. 29, no. 6, pp. 697–708, Nov. 2019.
